# The Novel Mouse Mutation *Oblivion* Inactivates the PMCA2 Pump and Causes Progressive Hearing Loss

**DOI:** 10.1371/journal.pgen.1000238

**Published:** 2008-10-31

**Authors:** Sarah L. Spiden, Mario Bortolozzi, Francesca Di Leva, Martin Hrabé de Angelis, Helmut Fuchs, Dmitry Lim, Saida Ortolano, Neil J. Ingham, Marisa Brini, Ernesto Carafoli, Fabio Mammano, Karen P. Steel

**Affiliations:** 1 Wellcome Trust Sanger Institute, Genome Campus, Hinxton, Cambridge, United Kingdom; 2 MRC Institute of Hearing Research, University Park, Nottingham, United Kingdom; 3 Venetian Institute of Molecular Medicine (VIMM), Padua, Italy; 4 Department of Biochemistry and Department of Experimental Veterinary Sciences, University of Padua, Padua, Italy; 5 Helmholtz Zentrum München, Institute of Experimental Genetics, Neuherberg, Germany; 6 Department of Physics, University of Padua, Padua, Italy; The Jackson Laboratory, United States of America

## Abstract

Progressive hearing loss is common in the human population, but we have few clues to the molecular basis. Mouse mutants with progressive hearing loss offer valuable insights, and ENU (*N*-ethyl-*N*-nitrosourea) mutagenesis is a useful way of generating models. We have characterised a new ENU-induced mouse mutant, Oblivion (allele symbol *Obl*), showing semi-dominant inheritance of hearing impairment. *Obl/+* mutants showed increasing hearing impairment from post-natal day (P)20 to P90, and loss of auditory function was followed by a corresponding base to apex progression of hair cell degeneration. *Obl/Obl* mutants were small, showed severe vestibular dysfunction by 2 weeks of age, and were completely deaf from birth; sensory hair cells were completely degenerate in the basal turn of the cochlea, although hair cells appeared normal in the apex. We mapped the mutation to Chromosome 6. Mutation analysis of *Atp2b2* showed a missense mutation (2630C→T) in exon 15, causing a serine to phenylalanine substitution (S877F) in transmembrane domain 6 of the PMCA2 pump, the resident Ca^2+^ pump of hair cell stereocilia. Transmembrane domain mutations in these pumps generally are believed to be incompatible with normal targeting of the protein to the plasma membrane. However, analyses of hair cells in cultured utricular maculae of *Obl/Obl* mice and of the mutant *Obl* pump in model cells showed that the protein was correctly targeted to the plasma membrane. Biochemical and biophysical characterisation showed that the pump had lost a significant portion of its non-stimulated Ca^2+^ exporting ability. These findings can explain the progressive loss of auditory function, and indicate the limits in our ability to predict mechanism from sequence alone.

## Author Summary

Progressive hearing loss is very common in the human population, but we know little about the causes. Environmental and genetic factors each may contribute. Knowledge of the genetic variants involved in hearing loss and understanding of the molecular and cellular mechanism of their action will aid the development of better treatments. One of the few genes known to be involved, in both mouse and humans, is *Atp2b2*, which encodes a calcium pump. We have discovered a new mutation in this gene leading to hearing loss in the mouse mutant oblivion. The mutation leads to a serine to phenylalanine substitution in a transmembrane domain. Mutations affecting such transmembrane domains are usually expected to interfere with the normal process of inserting the protein in the membrane and transporting it to its final destination on the plasma membrane of the cell. Surprisingly, the pump is produced and is targeted to the plasma membrane, in both cultured cells expressing the mutant gene and sensory hair cells from the oblivion mutant inner ear. However, we show it has impaired calcium pumping ability, which can account for the progressive hearing loss as well as the progressive degeneration of the sensory hair cells that we observe in the mutants.

## Introduction

PMCA2 is one of four isoforms of the plasma membrane Ca^2+^ pumps of mammalian cells [1],[2]. The expression of PMCA2 and PMCA3 is largely restricted to brain and muscle, whereas PMCA1 and 4 are ubiquitously expressed. PMCA2 and PMCA3 are more active in exporting Ca^2+^ than the ubiquitous isoforms [3], probably due to their higher affinity for the activator calmodulin. PMCA2, however, is peculiar in its very high activity even in the absence of calmodulin [4],[5]. In the ear, PMCA2 is expressed at high levels in outer hair cell stereocilia and apical membranes and at moderate levels in inner hair cell stereocilia and in the spiral ganglion [6]–[10]. It actively extrudes Ca^2+^ that has entered the hair cell during mechanoelectrical transduction [11]. This maintains the low intracellular levels of Ca^2+^ and may create a relatively higher concentration of Ca^2+^ in the endolymph surrounding the stereocilia, contributing to the maintenance of the electrochemical gradient needed for transduction to occur [11]. Bulk concentration of Ca^2+^ in mammalian cochlear endolymph is estimated at ∼20 µM [12]. PMCA2 is also important in maintaining sufficient extracellular Ca^2+^ in the vestibular system for the formation of the otoconia, the calcium carbonate crystals needed for sensing gravity and acceleration [13].

Transcripts for PMCA2 undergo alternative splicing at two sites. Site A is closer to the N-terminus and site C closer to the C-terminus. In the PMCA2 variant expressed in stereocilia [14],[15], the splicing introduces three exons at site A, generating variant *w*, and two alternative exons at site C, generating variant *a*. The C-site insert leads to a truncated pump that contains only about half of the original calmodulin binding domain [4],[5]. The doubly inserted *w/a* variant, seen in stereocilia, has an unusually limited ability to increase activity rapidly when challenged with a Ca^2+^ pulse, but has about the same high non-stimulated activity as the full-length *z/b* variant [16].

The Oblivion (*Obl*) mutant was identified as a new mouse mutant with progressive hearing loss from a large scale ENU mutagenesis screen [17]. The aim of this screen is to provide new models for deafness, especially progressive deafness which is common in the human population, and to identify the genes and underlying pathology in these new mutants. Here we report that the progressive hearing loss in *Obl* is due to a missense mutation in the gene *Atp2b2*, encoding PMCA2. We describe the hearing impairment and hair cell pathology in the mutants, the dysfunction of Ca^2+^ export by the mutated PMCA2 pump cloned and overexpressed in model cells and in cultures of utricles from the mutant mice.

## Results

### Progressive Hearing Loss in Oblivion Mutants

*Obl/+* heterozygotes have a normal Preyer reflex at one month old, but by two months only 58% offspring from *Obl*/+×+/+ matings showed a Preyer reflex (Table S1), suggesting progressive hearing loss in *Obl/+* mice. No vestibular defect, indicated by head-tossing or circling behaviour, was seen in these heterozygotes, although no detailed analysis of vestibular function was performed.

**Figure 1:**
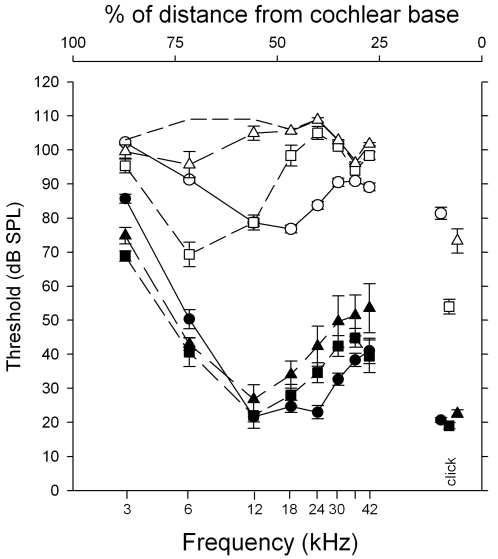
Auditory brainstem response thresholds in *Oblivion* heterozygous and wildtype animals. *Obl/+* mutants show significantly raised auditory brainstem response (ABR) thresholds compared to wildtype mice at P20 (circles), P59–62 (squares), and P89–91 (triangles). Mean ABR thresholds with standard error bars are given for +/+ mice (filled symbols) and *Obl/+* mice (open symbols). The dotted line without symbols indicates the maximum dB SPL output for the sound system at each frequency. The position along the length of the cochlear duct that best responds to each frequency (represented by % of total distance from the base) is indicated by the frequency place map at the top of the graph. Mean click ABR thresholds are plotted at an arbitrary point on the frequency axis; the position does not relate to the frequency content of the click stimulus.

To measure auditory thresholds, auditory brainstem responses (ABR), a reflection of cochlear and brainstem neural activity, were recorded in P20, P59–62 and P89–91 mice on their original C3HeB/FeJ genetic background (Figure 1). ABRs of wild-type mice showed an improvement in thresholds below 12 kHz from P20 to P59–62, perhaps indicative of maturation of the auditory system. From P20 to P89–91, wild type mice showed mild and progressive elevations of thresholds above 12 kHz.

*Obl/+* mice demonstrated a severe and age-related progressive hearing loss. *Obl/+* mice showed significantly raised thresholds at all frequencies, compared to age-matched wild-type controls (t-test, p<0.05), of up to 60–70 dB or more. In contrast to the Preyer reflex tests, even at P20 *Obl/+* showed large threshold elevations. At P59–62, the heterozygotes showed further threshold elevations which were most severe at higher frequencies, above 18 kHz. By P89–91, high frequency losses were compounded by severe losses across the entire range measured. This indicated a progressive hearing loss in *Obl/+* mice. The improvement of low frequency (3–6 kHz) ABR thresholds between P20 and P59–62 may indicate maturation of the developing auditory system between these ages.

*Obl/Obl* homozygous mutants show a very severe hearing and vestibular phenotype and are significantly smaller (10.5 g, SD 2.02) than age and sex matched *Obl*/+ littermates (17.3 g, SD 2.11; *t*-test, p<0.05, 31–34 days old). They (a) fail to develop a Preyer reflex; (b) fall from side to side whilst walking; (c) are unable to right themselves; and (d) curl towards their belly when lifted by their tail and do not display a reaching response when lowered towards a surface. Homozygotes also show hind limb stiffness and appear ataxic, which are not general features of vestibular dysfunction.

### Structure of the Inner Ear

**Figure 2:**
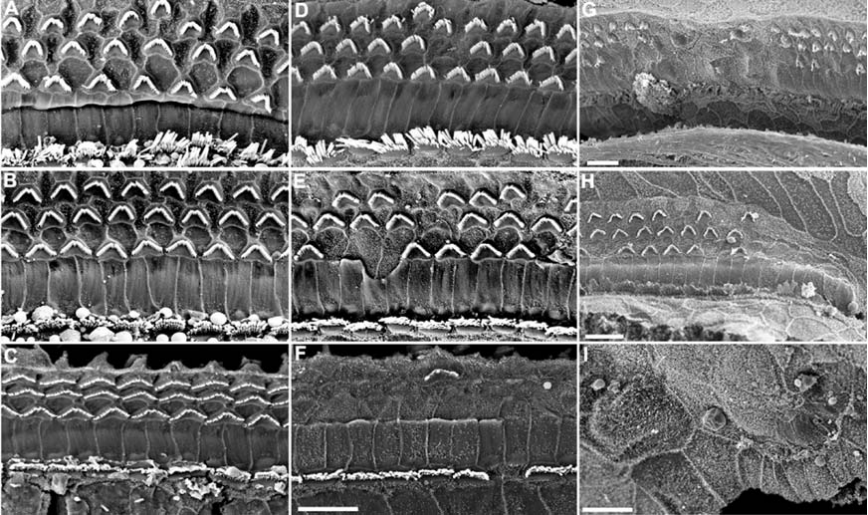
Analysis of wildtype, *Obl/+*, and *Obl/Obl* organ of Corti by scanning electron microscopy. At 3–4 months, normal animals show three rows of outer hair cells (OHC) and one row of inner hair cells (IHC) in the apex (A), middle (B), and base (C) of the cochlea. *Obl/+* have extensive OHC loss and some IHC loss in the base of the cochlea (F) and a few missing OHC in the middle of the cochlea (E). The apex appears normal (D). At 1 month of age, the phenotype in the *Obl/Obl* mice is extremely variable. In some regions of the base, middle and apex, the phenotype is similar to that seen in *Obl/+*. However, in other parts of the apex (G) and middle (H) regions of the cochlea, there are missing patches of OHCs. In some regions of the base, there is a complete degeneration of the organ of Corti, with no IHC, OHC, or supporting cells such as pillar cells present (I). Scale bar = 10 µm.

**Figure 3:**
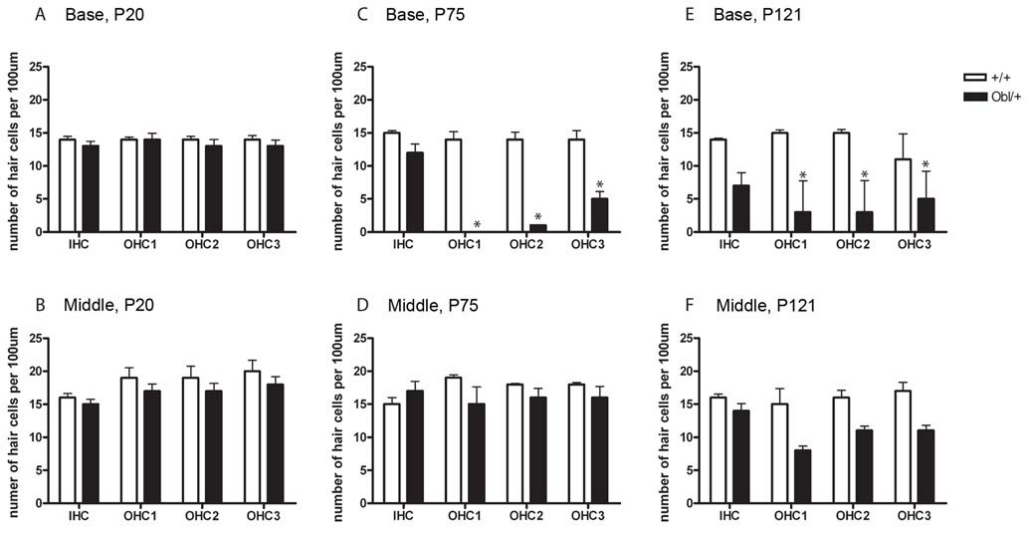
Hair cell counts in *Oblivion* heterozygous and wildtype animals. Hair cell counts show that outer hair cell loss in heterozygotes (*Obl/+*) at P75 is present in the base (C) but not the middle (D) of the cochlea. By P121, outer hair cell loss is seen in the base (E) and the middle (F) of the cochlear duct. IHC loss is seen in the base of the cochlea only at later ages (E). This suggests that hair cell loss in *Obl/+* mutants progresses in a base to apex direction, with OHC loss occurring first and IHC loss later. No OHC or IHC loss was seen in the base (A) or middle (B) regions of the cochlea at P20. Hair cell counts calculated as mean number of hair cells per 100 µm at P20 (+/+ n = 4–7, *Obl/+* n = 7), P72–75 (+/+ n = 4, *Obl/+* n = 5) and P121 (+/+ n = 3–4, *Obl/+* n = 4–5). Error bars indicate 95% confidence interval, T-test, * P-value<0.05.

The gross morphology of the middle ear ossicles and inner ear appeared normal in Oblivion mutants, both heterozygotes and homozygotes. Scanning electron microscopy in *Obl*/+ mutants at 3–4 months of age showed degeneration of hair cells, with the basal turn more severely affected than the apex, and outer hair cells (OHCs) more affected than inner hair cells (IHCs), a pattern that is commonly reported in damaged cochleas (Figures 2 and 3). *Obl/Obl* homozygotes were more severely affected than heterozygotes. However, there were many remaining hair cells with relatively normal appearance in the mutants, including a W-shaped arrangement of stereocilia, especially in the apical turn. Stereocilia fusion was seen in some, an early indicator of hair cell degeneration. At P20, no significant hair cell loss was detected in *Obl/+* mutants compared to their littermate controls (Figure 3A and 3B), despite the fact that we saw significantly raised ABR thresholds in another cohort of P20 heterozygotes (Figure 1). Hair cell counts from the basal and middle turns at P75 showed no significant OHC degeneration in the middle turn and no significant IHC loss throughout the cochlea in *Obl*/+ heterozygotes (Figure 3C and 3D). By P121, there was significant OHC and IHC loss in basal and middle turns in *Obl*/+ (Figure 3E and 3F). This suggests that the hair cell loss seen in these mutants is a secondary consequence of the hair cell not functioning correctly, rather than being the primary cause of raised thresholds in *Obl/+* mutants.

In *Obl/Obl* mutants at P30 there was highly variable hair cell degeneration, both within and between animals. In some regions there was scattered hair cell loss with a pattern similar to that seen in heterozygotes (Figure 2G and 2H), while in some regions towards the base there was complete degeneration of the organ of Corti with a complete absence of specialised cells, including supporting cells such as pillar cells (Figure 2I).

### Mapping and Identification of the Oblivion Mutation

**Figure 4:**
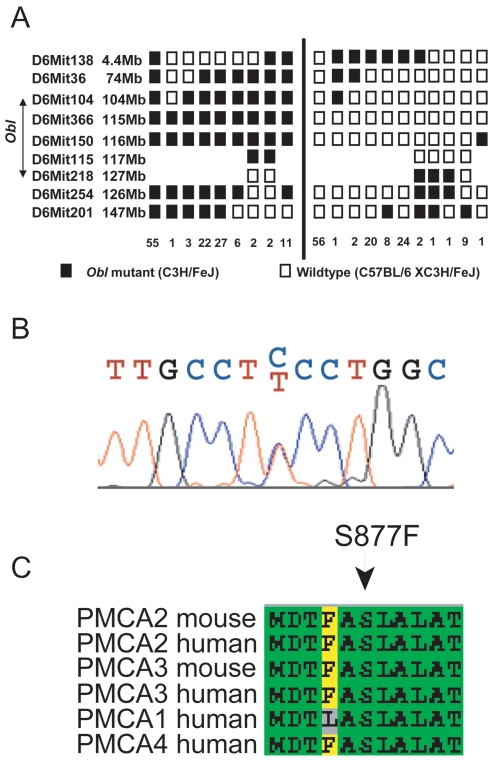
*Oblivion* chromosomal location, mutation, and PMCA2 sequence alignment. (A) *Obl* mutation maps to a 16 Mb region on mouse Chromosome 6, between *D6Mit104* and *D6Mit218*. Each box represents a marker typed, and each chromosomal arrangement detected is shown, with the number of animals possessing that chromosome given underneath. If a marker was not typed, then no box is present. (B) Sequence of *Atp2b2* in *Obl/+* mouse showing the C→T mutation and predicted sequence. (C) Sequence alignment of several PMCA family members showing Ser877 is highly conserved.

*Obl/+* mutants on a C3HeB/FeJ background were outcrossed to C57BL/6J and mutant F1 progeny were backcrossed to the original C3HeB/FeJ strain. Backcross litters were assessed for absence of a Preyer reflex and a genome-wide scan was performed on their DNA using 60 polymorphic microsatellite markers. We identified a region of linkage on chromosome 6 (Figure 4A) between markers *D6Mit104* and *D6Mit218*, corresponding to a physical distance of 16 Mb. This region contained a good candidate gene: *Atp2b2*. Genomic DNA was used to sequence the 19 coding exons of the gene, including the splice sites. We identified a C/T heterozygous peak in *Obl/+* mutants, suggesting a C→T transition (2630C→T) in exon 15 of *Atp2b2*, predicted to change a serine to a phenylalanine (S877F; Figure 4) in the mutant allele. This change was also confirmed by a restriction test assay that was used to genotype the colony. This assay was used to screen 19 inbred strains for the *Obl* mutation and none were found to have it, suggesting that it is not a common polymorphism. We found non-complementation of *Obl* with the deafwaddler mutant allele, *Atp2b2^dfw^*, confirming that the missense mutation we found was the pathogenic mutation (see Text S1).

### Functional Analysis of the Mutated PMCA2 Pump Overexpressed in Model Cells

**Figure 5:**
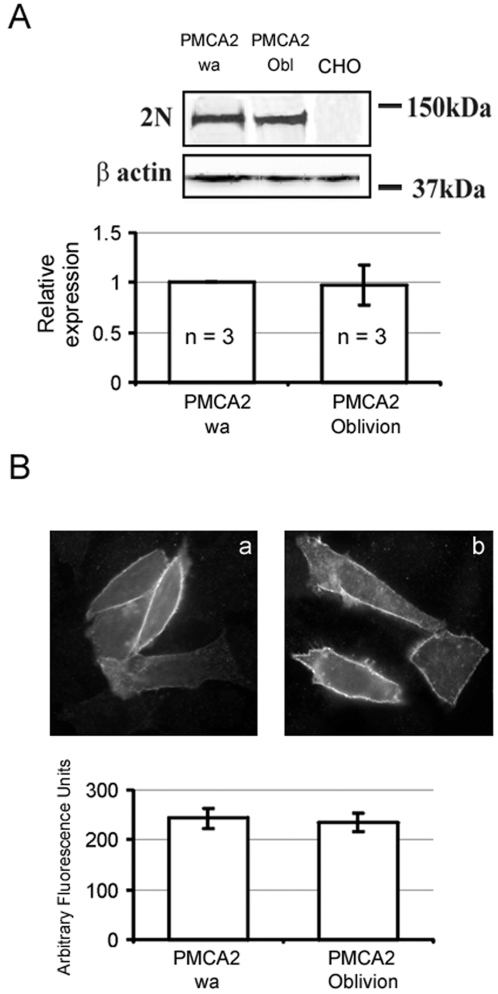
Expression and immunolocalization of recombinant PMCA2 pumps in CHO cells. (A) Western blotting analysis. Upper panel, 48 h after transfection CHO cells were washed twice with cold PBS buffer and scraped in lysis buffer (Tris-HCl 10 mM, EDTA 1 mM, PMSF 2 mM and DTT 1 mM). After centrifugation at 1000× g for 5 min, the cells were resuspended in 80 µl of lysis buffer and subjected to three cycles of freeze and thaw. The proteins of the lysates were quantified using the Bradford Reagent (Sigma-Aldrich). 15 µg of proteins were loaded on 10% polyacrylamide gel and transferred to nitrocellulose membranes which were incubated with polyclonal PMCA2 antibody 2N and monoclonal β-actin antibody (Sigma-Aldrich). After incubation with HRP-conjugated secondary antibodies (Santa Cruz Biotechnology, Inc., Santa Cruz, CA), the blots were developed with ECL reagents (Amersham Life Science). The band of ∼130 kDa corresponds to PMCA2 and the band of ∼42 kDa to β-actin. Lower panel, the relative expression of *wt* and *Obl* PMCA2 was determined by scanning the immunoblots digitally with Kodak 1D Image Analysis Software. The amount of Ca^2+^ pump protein was normalized for β-actin and the quantity of proteins was compared. (B) Immunolocalization of *wt* and *Obl* PMCA2 in transiently transfected CHO cells. The interaction with 2N antibody was revealed by the AlexaFluor488-conjugated secondary antibody. Upper panel, plasma membrane pattern of the overexpressed pumps (details in Materials and Methods). (a) wild type *w/a* pump. (b) *Obl/Obl* pump. Lower panel, fluorescence level in the plasma membrane quantified as described in Material and Methods. The SDs are indicated by the bars.

**Figure 6:**
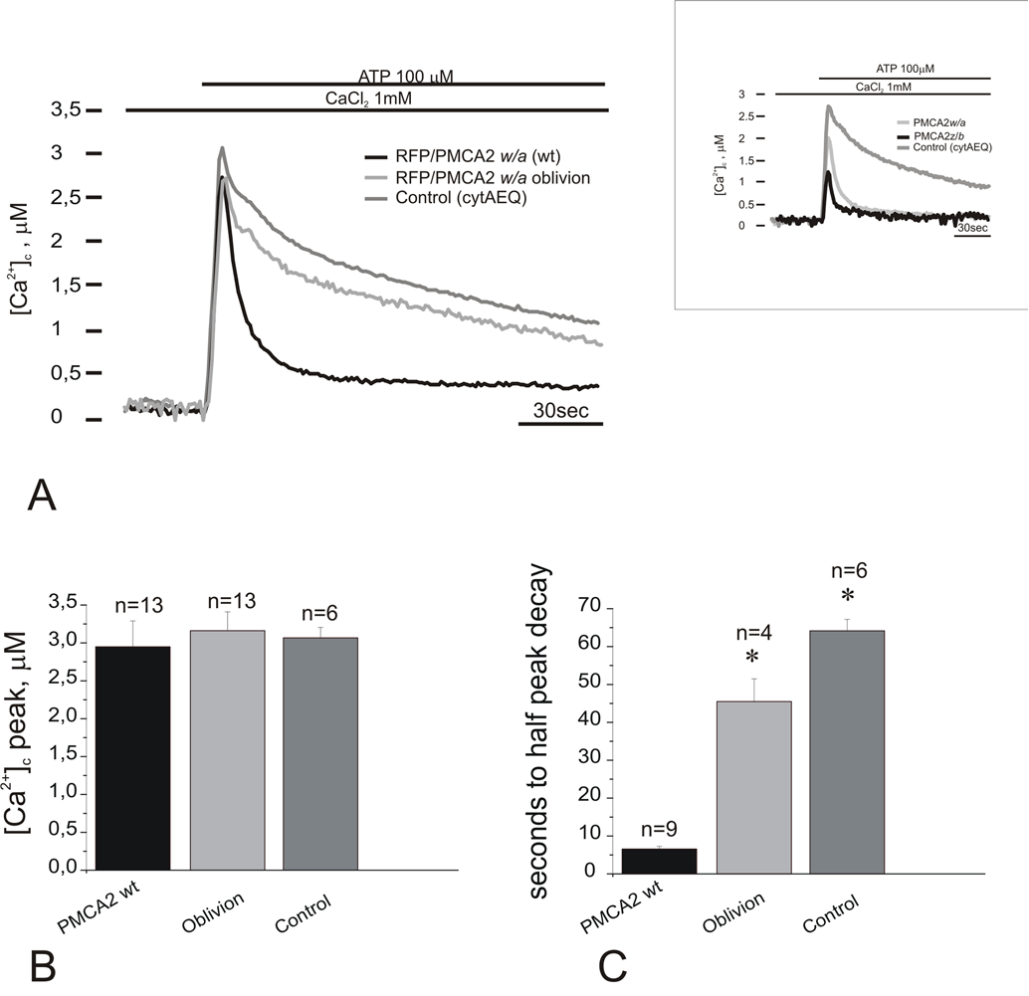
Activity of recombinant PMCA2 pumps in CHO cells. Cells were transiently co-transfected with the PMCA2 variants and cytAEQ, or only with cytAEQ (control). They were then perfused with KRB supplemented with 1 mM CaCl_2_. 100 µM ATP was used to produce a transient Ca^2+^ increase (A). The histograms in (B) and (C) show the means±SD of Ca^2+^ peaks and the half peak decay times, respectively (n indicates the number of experiments considered for the statistical analysis), * p<0.01 calculated with respect to the wt pump.

To investigate the effects of the serine to phenylalanine change on the functionality of the pump, mammalian expression plasmids for the S877F and the wild type variant of the PMCA2 pump were prepared and expressed in CHO cells. Appropriate controls (Western blotting and quantitative immunocytochemistry) established that the two pump variants were expressed at about the same levels, and were correctly delivered to the plasma membrane (Figure 5). CHO cells were transfected with the Ca^2+^ sensitive photoprotein aequorin (cytAEQ, [3]) and stimulated with ATP, an agonist of purinergic P2Y receptors that produces InsP_3_ generating a cytosolic Ca^2+^ transient. Under the experimental conditions, the height of the Ca^2+^ peak, and the kinetics of the return of the Ca^2+^ transient to baseline were controlled primarily by the PMCA pump: the much larger amounts of the overexpressed PMCA2 pump overshadowed the endoplasmic reticulum Ca^2+^ pump (SERCA) (see [16]) and the contribution of plasma membrane Ca^2+^ influx channels opened by emptying of intracellular stores to the shaping of the Ca^2+^ trace was disregarded, as their effect would be the same in the wild type and *Obl* measurements. The overexpressed *Obl* pump did not further depress the limited ability of the wild type *w/a* pump to control the height of the Ca^2+^ peak (Figure 6). The mutation, however, severely affected the resting activity of the pump that drove the return of the Ca^2+^ trace to baseline after the peak. The half time of the declining phase was 64.15±3.02 sec (n = 6) in control, 6.55±0.72 sec (n = 9) in the wild type *w/a* variant and 45.50±5.97 sec (n = 4), p<0.001, in *Obl*.

### Ca^2+^ Dynamics in the Hair Cell Stereocilia of Wild Type, *Obl/+*, and *Obl/Obl* Mice

**Figure 7:**
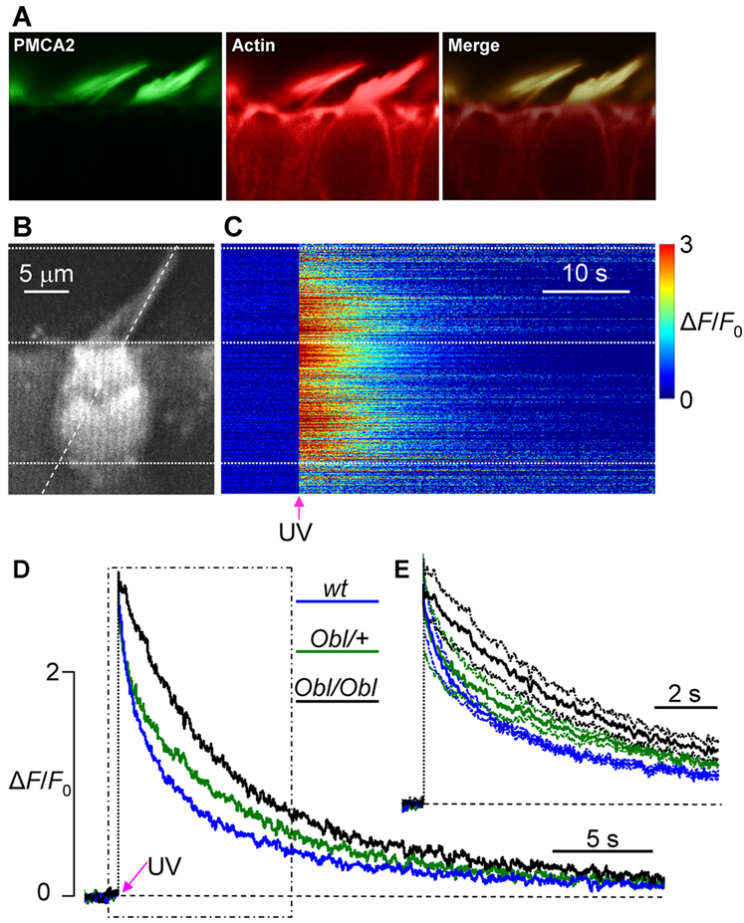
Dissipation of Ca^2+^ transients in utricular hair cells co-loaded with Fluo-4 and caged Ca^2+^. (A) Immunolocalization of the PMCA2 pump in the stereocilia of *Obl/Obl* utricular hair cells (details in Materials and Methods). Localisation in wildtype hair cells appears identical to that of mutants. (B) Confocal image of baseline Fluo-4 fluorescence in an organotypic culture of mouse utricular macula. The diagonal dashed line represents the scan line during subsequent data acquisition. The hair bundle comprises the top and middle horizontal dotted lines (the latter crosses the cell cuticular plate). The cell soma comprises middle and lower lines. (C) In this line-scan image, ordinates are pixel positions along the scan line, abscissa is time and fluorescence transients, Δ*F*/*F*_0_, evoked by a 4 ns UV pulse (arrow), are color-coded according to the color scale-bar at right. Signals from pixels below the lowermost horizontal dotted line arise from the cell adjacent to the hair cell in (B). (D) Fluorescence traces obtained by spatially averaging line-scan pixel signals within the stereociliary compartment of wild type (*wt*, blue solid line), heterozygous (*Obl/+*, green solid line), and homozygous Oblivion mice (*Obl/Obl*, black solid line). Each trace is the population average of n = 6 cells (for *wt* and *Obl/Obl*) or n = 5 cells (for *Obl/+*). The region within the dash-dotted box is shown on a faster time scale in (E). Also shown in (E) are confidence intervals (dash-dotted lines) for the average Δ*F*/*F*_0_ responses (solid lines).

**Figure 8:**
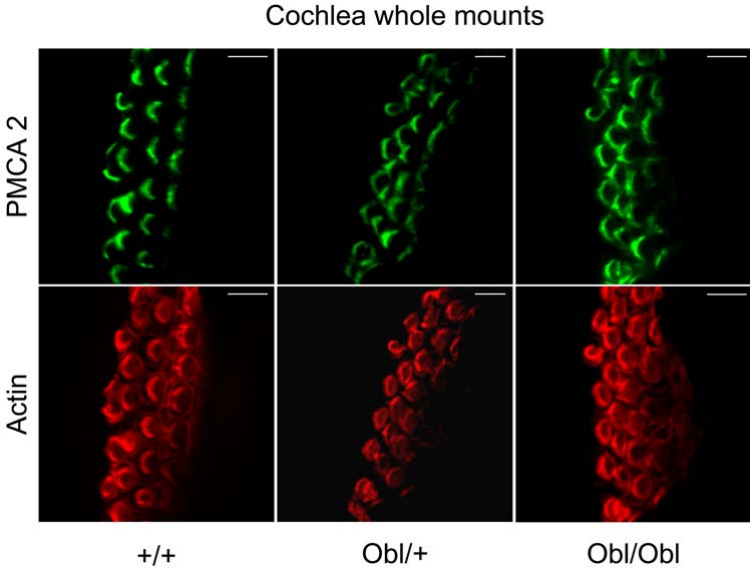
Immunolocalisation of PMCA2 in cochlear hair cells. Confocal imaging immunoassays of PMCA2 (green) in cochlear whole mounts from wild type (+/+), heterozygous (*Obl*/+), and homozygous (*Obl/Obl*) mice (red, actin). For details, see Materials and Methods.

To characterize Ca^2+^ dynamics in the stereocilia of hair cells, cultures of immature utricular maculae were obtained from wild type and mutant mice (see Materials and Methods). Ca^2+^-dependent changes in fluorescence evoked by the photorelease of intracellular caged Ca^2+^ (4 ns single UV pulse) were monitored with a temporal resolution of 6 ms using confocal laser scanning microscopy. Immunofluorescence labelling with isoform specific antibodies showed that PMCA2 was correctly located in the stereocilia of homozygous mutant organotypic cultures of utricular maculae (Figure 7A) and organ of Corti (Figure 8). Figure 7B shows a macular hair cell loaded with Fluo-4. Fluorescence changes in the stereocilia were monitored repeatedly by a line-scan positioned along the hair bundle and extending into the cell soma (dashed line). Time-dependent post UV pulse changes in fluorescence at different parts of the line scan are illustrated in Figure 7C. The time course of the stimulus-evoked changes in fluorescence (Δ*F*), normalized to basal (pre-stimulus) fluorescence (*F*_0_), are compared for wild type (*wt*, blue trace) heterozygous Oblivion mice (*Obl/+*, green trace) and homozygous Oblivion mice (*Obl/Obl*, black trace) in Figure 7D. The time courses matched well at the peak, although the [Ca^2+^]_i_ transient decayed more slowly for the *Obl/Obl* mice. To highlight the differences, the traces are re-plotted in Figure 7E on an expanded time scale (solid lines), together with their respective confidence intervals (dash-dotted lines). A single exponential fit to the first 10 s of these transients yielded significantly longer decay time constants for the *Obl/+* and *Obl/Obl* cultures: τ*_wt_* = 2.8±0.4 s for *wt*, τ*_Obl/+_* = 4.2±1 s for *Obl/+* mice (p = 0.02, if compared to *wt* using the ANOVA test) and τ*_Obl/Obl_* = 6.9±1.3 s for *Obl/Obl* mice (p<0.01). No significant divergence in the early phase (first 2 s) of the decay was found for *Obl/+* mice relative to *wt*, consistent with the lack of evident phenotypic vestibular defects in these mice. At later times, the decay tended to diverge possibly due to the contribution of complex processes such as Ca^2+^-induced Ca^2+^ release. These contributions were not investigated further.

## Discussion

Progressive, age-related hearing loss affects 60% of humans over the age of 70 [18]. The condition is a multifactorial disorder to which genetic variation, disease and environmental influences such as acoustic trauma are all contributing factors, making identification of the genes involved in humans difficult. The mouse is an ideal resource to study the genetics of progressive hearing loss due to the possibility of controlling both genetic background and the environment.

The present study has identified a new ENU-induced allele of *Atp2b2*, *Atp2b2^Obl^* in the *Obl* mouse mutant. This allele contains a C to T missense mutation in exon 15 of *Atp2b2*, causing a non-conservative amino acid substitution of serine by phenylalanine in transmembrane domain 6 of the PMCA2 pump [1],[19]. The serine at position 877 is highly conserved in both human and mouse and also between other members of the PMCA family (Figures 4C and S2). The S877F mutation is of special interest on at least two accounts. One is the finding that substitutions in the transmembrane residues of the PMCA pump frequently impair its correct plasma membrane targeting [20]. However, the mutant *Obl* pump was correctly delivered to the plasma membrane in both the model cells and in native stereocilia. In another *Atp2b2* mutant allele, Wriggle mouse Sagami (*wri*), a missense mutation in transmembrane domain 4 completely abolished the expression of PMCA2 protein in stereocilia of cochlear hair cells [21]. The second reason for interest stems from studies of the SERCA pump of endoplasmic reticulum, considered a model for all P-type pumps. Conserved residues within transmembrane domain 6 of SERCA have been shown to be components of the channel through which Ca^2+^ is translocated [22] and are also present in the PMCA pump. It is pertinent to quote at this point an earlier study on some point mutations in transmembrane domain 6 of another isoform of the pump, PMCA4 [23]. In that study, one of the mutants (S877A) appears to have higher activity than the wild type pump. The reason for the different effect of the S877A mutation of PMCA4 and the S877F mutation of PMCA2 that we report here is not clear. However, the PMCA4 study is difficult to compare with the present one, since it analyzed the pump in crude microsomes of overexpressing COS cells, and offered no information on the localization of the expressed pump in the plasma membrane.

The *Obl* mutation is the seventh mutation in mouse *Atp2b2* to be reported. In the deafwaddler (*dfw*) mouse a missense mutation in *Atp2b2* leads to a partial loss of function [8] and PMCA2 retains 30% of its Ca^2+^ pumping activity [24]. A spontaneous T692K mutation in *Atp2b2* led to clear ataxic behaviour, with normal mRNA levels, in a second unnamed mutant [25]. A further four mutations of mouse *Atp2b2* have been described: deafwaddler 2J (*dfw^2J^*), deafwaddler 3J (*dfw^3J^*), wriggle mouse sagami (*wri*) and a targeted null mutation. Analysis of mRNA transcripts and protein levels suggests that these latter four mutations are all null alleles [13],[21],[26]. Homozygotes for these alleles show severe ataxia by 10 days of age as well as profound deafness. The profound deafness and ataxia detected in *Obl* homozygotes is similar to the phenotype of these other known *Atp2b2* null mutants [13],[26],[27]. Non-complementation between *Obl* and *dfw* confirmed that these two mouse mutants are likely to be allelic. The mutations identified in the *Atp2b2* gene are shown in Figure S1.

Both the *Obl* and the *dfw* mutations significantly reduce the non-activated Ca^2+^ exporting ability of the PMCA2 protein. Observations on organotypic cultures showed that the defect of the pump observed in model cells also reduced its ability to remove the Ca^2+^ produced by UV photolysis in stereocilia. Thus, the *Obl* mutant has lost a significant portion of the non stimulated, longer-term Ca^2+^ exporting ability in respect to the *w/a* PMCA2 pump.

In the present work we show that hearing loss in *Obl/+* mice is detected at P20 and progresses in its severity with increasing age. Threshold shifts of up to 60-70 dB were found at frequencies corresponding to the basal and middle turns of the cochlea, where the majority of outer hair cell loss was detected. Analysis of *Obl/+* mice at P20 showed no OHC or IHC loss despite the fact that they had a significant hearing impairment, confirming the previous suggestion that although hair cells are present they are not functioning normally [28].

IHC loss was detected in the base and middle of the cochlea in *Obl/+* mutants at 4 months of age. The early degeneration of the OHCs seen in heterozygous *Obl* (and *dfw*) mice, leading to complete degeneration of the organ of Corti in the base of the cochlea, is similar to that seen in some human patients with age-related hearing loss [29],[30]. It is not clear why hair cell dysfunction leads to hair cell degeneration in these mouse mutants, but prolonged abnormal calcium homeostasis may contribute to hair cell death. In *dfw* mice at approximately P60, distortion product otoacoustic emission studies show that PMCA2 is important for the correct functioning of outer hair cells, especially at high frequencies [31]. Mice expressing the *Atp2b2^dfw2J^* allele, [9] demonstrated a lowered endocochlear potential and reduced endolymphatic calcium concentration, and thus have a reduced capacity for mechanoelectrical transduction. Taken together, these features may explain why ABR thresholds in *Obl/+* mice are elevated above controls, but are still recordable. An interesting observation from the ABRs recorded in P59–62 and P89–91 *Obl/+* mice is that click thresholds are more sensitive than the best tone threshold, by a factor of 15–22 dB. Clicks produce a more synchronised activation of a wider region of the basilar membrane compared to tone pips, and this may produce a summation of activity, reflected in lower click-evoked ABR thresholds.

When *Obl* was placed on a mixed C3HeJ/FeB and C57BL/6J background, the progressive hearing loss in heterozygotes seemed more severe (data not shown). Onset and severity of progressive hearing loss seen in heterozygotes of other *Atp2b2* mutant alleles have been shown to vary considerably depending on the genetic background on which the mutation arose [13],[21],[26],[28],[32]. This is due to the presence of modifier alleles, one of which has been identified as the G753A variant of *Cdh23* at the *ahl* locus (also known as modifier of deafwaddler, *mdfw*) [32],[33]. Interactions between heterozygous *Atp2b2* mutations and *Cdh23* mutations have been shown to worsen the progressive hearing loss seen in some human patients. Heterozygous mutations (*ATP2B2^V586M^*) increase the severity of the progressive hearing loss seen in human patients with mutations in *CDH23* [34]. The *ATP2B2^V586M^* mutation reduced the level of PMCA2 protein produced by 50%, although on its own it was not sufficient to cause hearing impairment in humans [34]. More recently, hearing loss has been reported in a human patient with an *ATP2B2^G293S^* mutation and a *CDH23^T1999S^* mutation. The parents of the patient carried either the *ATP2B2* or the *CDH23* mutation, but had no hearing impairment [16].

The *ATP2B2* gene has recently been implicated in the deafness in 3p- syndrome. The syndrome is characterised by developmental delay, growth retardation and craniofacial abnormalities (see [35]), which is sometimes, but not always, associated with a severe sensorineural hearing loss. In 3p- syndrome patients with a hearing loss, a deletion in the 3p25.3 locus was mapped to a region containing 18 genes including *ATP2B2*. It is likely that haploinsufficiency of *ATP2B2* is responsible for the deafness associated with this syndrome [36].

As progressive hearing loss is so common in the human population and we know so little about its molecular basis, identifying other mutations in and modifiers of the *Atp2b2* locus in mouse inbred strains may be of importance in identifying new loci involved in progressive and age-related hearing loss in humans.

## Materials and Methods

### Mice

The founder mouse carrying the Oblivion mutation (gene symbol *Obl*, original identifier DEA14) arose from the ENU mutagenesis program at Munich [17]. Mutations were generated by injecting 3 doses of 80–90 mg/kg bodyweight of *N*-ethyl-*N*-nitrosourea (ENU) into C3HeB/FeJ males. F1 progeny were screened for a range of phenotypes, including deafness and balance disorders. The founder *Obl/+* mutant was identified by the absence of a Preyer reflex at 3 months. The mutant colony was maintained on the C3HeB/FeJ background, and all ABR and structural analysis was carried out on this genetic background. The care and use of animals was carried out in accordance with UK Home Office regulations and the Animal Care and Use Committee of the University of Padua.

### Inner Ear Clearing and Ossicle Dissection

Half heads were fixed in Bodian's fixative and cleared with glycerol using a standard protocol. The inner ear was examined for signs of malformation. Middle ear ossicles were dissected out and studied. Six *Obl/+* mutants and six littermate controls between 3–4 months age were analysed.

### Scanning Electron Microscopy

Inner ears were fixed in 2.5% glutaraldehyde in 0.1 M sodium cacodylate buffer, the organ of Corti was exposed and samples were processed using the osmium tetroxide-thiocarbohydrazide (OTOTO) method [37]. After critical point drying and sputter coating with gold, samples were examined using a Phillips XL30 scanning electron microscope at 10 kV or a Hitachi S4800 FE Scanning Electron Microscope at 5 kV. Initial characterisation was performed on 3 *Obl/+* and 3 littermate controls at 3–4 months of age. Hair cell degeneration was assessed in the basal turn (20–30% of the total distance from the base of the cochlear duct) and middle turn (55–65% from the base) at 20 (P20), 72–75 and 121 days old. Between 3 and 7 *Obl*/+ and +/+ mice were examined at each age. Hair cells with intact stereocilia bundles were counted over a stretch of at least 200–300 µm of the cochlear duct. Hair bundles that were damaged or showed fusion were still counted as being present. A two tailed T-Test was performed on hair cell counts for each hair cell row in each region at 95% confidence intervals, with the number of cases and standard deviations included in the analysis. A two tailed T-test was carried out on the weights of *Obl/Obl* mutants compared to littermate (*Obl/+*) controls in the same way.

### Preyer Reflex and Auditory Brainstem Response (ABR)

The Preyer reflex (pinna flick) was detected using a custom-built click box to deliver a calibrated 20 kHz sound burst at 90 dB SPL. Up to 88 mice from *Obl/+*×*+/+* matings were tested weekly from 3 to 8 weeks of age, although the numbers of mice at each time-point varied. For ABR recordings, a separate cohort of mice were anaesthetised (urethane 2 mg/g) and subcutaneous needle electrodes inserted on the vertex (active), and over the left (reference) and right (ground) bullae. A calibrated sound system was used to deliver free-field click (0.01 ms duration) and tone pip (various frequencies from 3–42 kHz of 5 ms duration, 1 ms rise/fall time) stimuli at a range of intensity levels in 3 dB (or multiple) steps. Averaged responses to 512 stimuli, presented at 21.1 s^−1^, were analysed and thresholds established as the lowest sound intensity giving a visually-detectable ABR response. ABR recordings were obtained from a total of 61 mice, 28 at P20 (*+/+*, n = 9; *Obl/+*, n = 19), 14 at P59–62 (*+/+*, n = 5; *Obl/+*, n = 9) and 19 at P89–91 (*+/+*, n = 5; *Obl/+*, n = 14).

### Mapping the *Obl* Mutation

*Obl/+* mutants on a C3HeB/FeJ background were outcrossed to C57BL/6J wild type females. *Obl/+* F1 progeny were then backcrossed to +/+ animals from the original C3HeB/FeJ strain. Offspring from these backcross matings were examined at 2 months of age or older (as this is the age at which *Obl/+* mice on the original genetic background show profound hearing loss) using the Preyer reflex. Tail and pinna tissue were collected for DNA preparation. A total of 255 backcross mice were analysed (129 *Obl/+*, 126 +/+). A genome-wide scan was conducted with 60 microsatellite markers approximately 25 cM apart, that had been shown to be polymorphic between C3HeB/FeJ and C57BL/6J inbred strains (Table S2). Additional microsatellite markers used for fine mapping of the *Obl* mutation were: *D6Mit36*; *D6Mit104*; *D6Mit150*; *D6Mit115*; *D6Mit218*; *D6Mit254*. PCR was performed using standard techniques.

### Mutation Screening and Confirmation

Sequence analysis was performed on genomic DNA in *Obl/+* mutants and littermate controls using primers designed to amplify the coding exons and splice sites of the *Atp2b2* gene. The primer sequences are listed in Table S3. PCR was performed using standard techniques and products were cleaned using magnetic bead separation (Ampure) and sequenced using BigDye Terminator Cycle sequencing kit (Applied Biosystems). Sequence traces were analysed using Gap4 software [38]. To confirm the mutation identified in exon 15 and for genotyping of the colony, a PCR-based genotype test was designed. The 2630C→T missense mutation did not change a restriction enzyme recognition site, so primers were designed flanking the mutation site that would incorporate a *StyI* recognition site in the wildtype (C) allele, but not in the mutant (T) allele. Primers OblRTF (5′-CTT CTT CTC CCT GCC ACT GTC GTA G) and OblRTR (5′-CCA CCG AGA CAC CGG TCC CGG TTC) were used for PCR. The 111 bp PCR product was digested with *StyI* (New England Biolabs) which cuts the wildtype allele giving an 89 bp fragment while the mutant allele remains uncut. This genotyping tool was used to establish whether the sequence change in *Obl* DNA was a polymorphism in a total of 17 inbred strains: BALB/C, CBA/Ca, C3HeB/FeJ, DDY/Jc1, 129X1/SvJ, A/J, Bxd-1/Ty, C58/J, CE/J, DA/HuSn, DBA/2J, FL/1Re, LP/J, NON/LtJ, RBG/Dn, St/bJ and SWR/J.

### Cloning and Mutagenesis of pmRFP-PMCA2 w/a

mRFP was amplified from pCDNA3.1/zeo-mRFP (kindly donated by Dr. M. Zaccolo, Padua, Italy) using the following primers, forward: 5′-GC**GCTAGC**ATGGCCTCCTCCGAGGACGTCA-3′ and reverse: 5′-GC**AGATCT**GAGGCGCCGGTGGAGTGGCGG-3′, bearing restriction sites for NheI and BglII, respectively (in bold). The PCR product was then digested with NheI and BglII and inserted in pEGFP-c1 (Clontech, Palo Alto, CA) digested with NheI and BglII to create pmRFP-c1. PMCA2 *w/a* in pMM2 (kindly provided by Dr. Strehler, Rochester, MN) was excised by independent digestion with SalI-EcoRI and EcoRI-KpnI and inserted into XhoI-KpnI sites of pmRFP-c1 in a three-part ligation reaction resulting in pmRFP-PMCA2wa. The construct was controlled by sequencing. Site-directed mutagenesis was carried out to obtain the mutant cloned in the appropriate vector. pmRFP-PMCA2w/a was used as target and experiments were performed according to the manufacturer's standard protocol (Stratagene, Cedar Creek, TX) The following primers were used: *Obl* 5′ CATCATGGACACATTTGCTTTCCTGGCCCTGGCAACAGAGC 3′ (forward) and 5′ GCTCTGTTGCCAGGGCCAGGAAAGCAAATGTGTCCATGATG 3′ (reverse)

### Immunolocalization of the Expressed Pumps and Membrane Fluorescence Computation

CHO cells were grown in Ham's F12 medium, supplemented with 10% fetal calf serum (FCS). Before transfection, they were seeded onto 13 mm glass coverslips and allowed to grow to 50% confluence. Transfection with 3 µg of plasmid DNA (or 1.5 :1.5 µg in the case of co-transfection) was carried out with a Ca-phosphate procedure [39]. Immunocytochemistry quantified the expressed pump proteins in the plasma membrane of transfected cells. CHO cells expressing the PMCA2 variants, were stained with polyclonal isoform-specific PMCA antibody 2N (Affinity Bioreagent, Inc., Golden, CO) or a monoclonal antibody recognizing all pump isoforms (5F10, Affinity Bioreagent, Inc., Golden, CO), at a 1∶100 dilution in PBS. Staining was carried out with Alexa 488 labelled anti-rabbit or anti-mouse secondary antibodies (Molecular Probes, Invitrogen Corp., Carlsbad, CA) at a 1∶50 dilution in PBS. Cells were imaged on a spinning disk confocal microscope (Ultraview; Perkin-Elmer) by using a X60 oil-immersion objective at a N.A. (PlanAPo; Nikon, Tokyo, Japan). Regions of interest were selected by applying an edge-finding (Sobel) digital filter, thus limiting the analysis to plasma membrane areas. The total fluorescence intensity in membrane-delimiting regions of interest was quantified with software developed in our laboratory. For each construct fluorescence was averaged over a total of 50 cells in 3 different slides.

### Ca^2+^ Measurements with Recombinant Aequorin

Transfected cytAEQ were reconstituted by incubating CHO cells for 1–3 h with 5 µM coelenterazine in Dulbecco's modified Eagle's medium (D-MEM) supplemented with 1% FCS, at 37°C in a 5% CO_2_ atmosphere. Additions to the KRB medium (1 mM CaCl_2_, 100 µM ATP) were made as specified in the figure legends. The experiments and luminescence calibration into [Ca^2+^] values were carried out according to [40]. The experiments were terminated by lysing the cells with 100 µM digitonin in a hypotonic Ca^2+^-rich solution (10 mM CaCl_2_ in H_2_O) to discharge the remaining aequorin pool. Briefly, a 13-mm round coverslip with the transfected cells was placed in a perfused thermostated chamber in close proximity to a low-noise photomultiplier, with a built-in amplifier discriminator. The output of the discriminator was captured by a Thorn-EMI photon-counting board and stored in an IBM-compatible computer for further analyses. Luminescence was calibrated off-line into [Ca^2+^] values by using a computer algorithm based on the Ca^2+^ response curve of wt aequorin. Data are reported as mean±SD. Statistical differences were evaluated by Student's 2-tailed t-test for unpaired samples. A p value<0.01 was considered statistically significant.

### Preparation of Organotypic Utricle Cultures

To access utricular maculae of wild type or mutant mice between postnatal day 3 (P3) and P4, the otic capsule was opened medially and the endolymphatic compartment of the macula cut open. The otolithic membrane was removed after 15 min incubation in dissection saline to which 0.1 g/l bacterial subtilisin (type XXIV; Sigma-Aldrich, St. Louis, MO) had been added. Dissection saline was composed of Hank's Balanced Salt Solution (HBSS; part number H6648, Sigma-Aldrich) with 10 mM HEPES, 10.000 U/l penicillin and 25 µg/l fungizone. HBSS contained (in g/l): 0.4 KCl, 0.06 KH_2_PO_4_ (anhydrous), 0.35 NaHCO_3_, 8.0 NaCl, 0.048 Na_2_HPO_4_ (anhydrous), 1 D-glucose. The epithelium was fixed by Cell-Tak (BD Biosciences, Bedford, MA), mixed with 90% NaHCO_3_, to the lateral side of a glass capillary (1.5 mm diameter, 5 mm length), which had been previously glued to a microscope slide by a small drop of Sylgard Silicon Elastomer (Dow Corning, Wiesbaden, Germany). Cultures were preserved for one day at 37°C in a complete medium of 95% D-MEM/Ham's F-12 (1∶1) (concentration 1X, liquid form, containing L-glutamine but no HEPES; Gibco, Invitrogen Corp., Carlsbad, CA) and 5% fetal bovine serum.

### Immunolocalization of PMCA2 in the Stereocilia of Hair Cells in Utricle Cultures

Organotypic cultures dissected from P3 wild type and mutant mice pups were maintained over night at 37°C in D-MEM/Ham's F-12 (1∶1) medium with 5% fetal bovine serum. Tissue preparations were fixed in paraformaldehyde 4% for 20 min at room temperature, rinsed in washing solution (PBS containing BSA 2%) and permeabilised with washing solution containing Triton 0.1% for 1 h at room temperature. Incubation with primary PMCA antibody 2N was carried out overnight at 4°C using a 1∶100 dilution in washing solution. FITC-conjugated rabbit anti-IgG antibody (Invitrogen) was used as secondary antibody for pump detection (1∶200 dilution in washing solution, 2 h at room temperature). The preparation was mounted on a coverslip and imaged up side down on an inverted spinning disk confocal microscope (Ultraview; Perkin-Elmner) using a 60× oil-immersion objective at a 1.4 N.A. (PlanApo; Nikon, Tokyo, Japan).

### Whole Cochlea Immunohistochemistry

Cochleae dissected from P5 mice were fixed in 4% paraformaldehyde for 20 min at room temperature, rinsed in PBS containing 2% BSA (rinse solution) and permeabilized for 1 hour at room temperature with 0.1% Triton, dissolved in rinse solution. Tissues were stained with polyclonal isoform-specific PMCA antibody 2N (Affinity Bioreagent, Inc., Golden, CO) by incubation overnight at 4°C with specific polyclonal antibodies (2.5 µg/ml) (Invitrogen) diluted in rinse solution. A FITC conjugated rabbit anti–IgG antibody (5 µg/ml, Invitrogen) was used as secondary antibody, following incubation in rinse solution for 2 hours at room temperature. F–Actin was stained by incubation for 1 hour at room temperature with rhodamine phalloidin (7 µM, Invitrogen, R415) diluted in rinse solution. Stained samples were sandwiched between two coverslips and imaged with a 60× oil–immersion objective (NA 1.4, Plan Apo, Nikon, Tokyo, Japan) attached to an inverted microscope (Eclipse 200, Nikon) equipped with a Nipkow disk confocal scanning head (Ultraview, Perkin Elmner, USA). Confocal fluorescence images were captured with a scientific grade cooled CCD camera (Orca, Hamamatsu Photonics, Hamamatzu City, Shizuoka, Japan).

### Confocal Imaging of Organotypic Utricle Cultures

Cultures were loaded with 10 µM cell permeant Fluo-4 AM (Invitrogen) for 50 min at 37°C in D-MEM supplemented with 10 µM cell permeant NP-EGTA AM (Invitrogen), 25 µM sulfinpyrazone and Pluronic F-127 (0.1% w/v). For de-esterification, cultures were transferred to an experimental chamber mounted on the stage of a confocal imaging setup (Biorad Radiance 2100) incorporating an upright microscope (Eclipse E600FN, Nikon, Tokyo, Japan) and superfused for 20 min with a medium composed of HBSS supplemented with 4.4 g/l glucose and 2 mM anhydrous CaCl_2_ (pH 7.4, Osm 330). Experiments were performed with a 100× water-immersion objective (N.A. 1.00, LUMPlanFl, Olympus, Tokyo, Japan) using the same perfusion medium. Fluo-4 fluorescence was excited by the 488 nm line of an argon laser coupled by fiber optics to the confocal microscope. Fluorescence emission was selected around 528 nm using a narrow-band (50 nm) interference filter. Fluorescence images of utricle hair cells in the organotypic cultures were acquired with a resolution of 512×512 pixel by scanning at 512 lines per second under control of the Biorad Laser Sharp software. To be retained for subsequent recording, imaged cells had hair bundles extending for their entire length in a single confocal plane (bundle planarity condition), a possibility afforded by having the culture attached to a curved surface.

### Confocal Line-Scan Recording and UV Photolysis of Caged Ca^2+^ in Utricular Hair Cells

Dynamic fluorescence data were acquired in the ‘linescan’ mode to produce a scan series of fluorescence intensity values, *F*, measured in the photomultiplier tube PMT units from a value of 0 to a saturating value of 255. Laser intensity and PMT gain were adjusted to accommodate the dynamic range of changes in *F*. Typical background fluorescence values, measured from regions devoid of obvious cellular structures, were ∼1 PMT units, while pre-stimulus (basal) levels, *F*_0_, averaged over the entire length of the hair bundle, were ∼8 PMT units. In all experiments pinhole aperture was adjusted to the same value, yielding confocal section with 3 µm thickness. To assay the Ca^2+^ extrusion activity of the PMCAs, an area of ∼3000 µm^2^, comprising a few hair cells in the cultured utricular macula, was exposed to UV radiation generated by an air-cooled 337 nm pulsed nitrogen laser (Model VSL-337ND-S, Spectra Physics, Mountain View, CA, USA) connected to the microscope through a 600 µm ∅ optical fiber. UV light was directed onto the sample by reflection off a 400 DCLP dichromatic beam splitter (Chroma) positioned at 45° just above the microscope objective lens. A single laser pulse (4 ns) delivering a maximum of 326 µJ of energy (at the laser output) was used to photorelease Ca^2^ from the caged state (NP-EGTA bound to Ca^2+^) in the brief time interval between the 2500^th^ and the 2501^st^ scan line. In a typical record lasting about 120 s, 20 000 consecutive lines were acquired.

### Off-Line Analysis of Fluorescence Transients

All data were analyzed offline on a personal computer using the Matlab 7.0 (The MathWorks, Inc., Natick, MA) software environment. Data are presented as Δ*F*/*F*_0_ where Δ*F* = *F*−*F*_0_. In these expressions, raw pixel values are spatial averages along the hair bundle. Maximal percent fluorescence changes, (*F*_max_−*F*_0_)/*F*_0_, were about 280%. To estimate the slow time constant, *τ*, of recovery to baseline, transients peaking at Δ*F*_max_, were fitted by a single exponential function during the first 10 s from the UV pulse. Data are given as mean±standard error of the mean (S.E.M.).

## Supporting Information

Figure S1 Mutations identified to date affecting PMCA2 protein in mice. Three mutations are missense mutations leading to amino acid substitutions (*dfw*, *wri*, *Obl*), two are small deletions that lead to frame shift mutations and premature truncation of the PMCA2 protein (*dfw^2J^*, *dfw^3J^*), and one is a targeted null allele (*Atp2b2^tm1Ges^*) [8],[13],[21],[26]. Adapted from [21].
(0.29 MB TIF)

Figure S2 Amino acid alignments of PMCA isoforms. Alignment of the amino acids residues in transmembrane domain 6 of PMCA isoforms in different species (A) and of PMCAs and other P-type ATPases (B). The similarity analysis was performed using the ClustalW program. GenBank accession numbers are listed: NP_001674 (*Homo sapiens*), Q9ROK7 (*Mus musculus*), NP_036640 (*Rattus norvegicus*), XP_509257 (*Pan troglodytes*), NP_777121 (*Bos taurus*), NP_999517 (*Sus scrofa*), Q00804 (*Oryctolagus cuniculus*), AAK11272 (*Rana catesbeiana*), AAH77905 (*Xenopus laevis*), AAR28532 (*Procambarus clarkii*), AAR13013 (*Stylophora pistillata*), AAK68551 (*Caenorhabditis elegans*), XP_653525 (*Entamoeba histolytica*), AAP46286 (*Trypanosoma brucei*), EAL62716 (*Dictyostelium discoideum*), NP_849716 (*Arabidopsis thaliana*), AAB81284 (*Paramecium tetraurelia*), NP_001001323 (PMCA1), NP_068768 (PMCA3), NP_001675 (PMCA4), NP_004311 (SERCA1), NP_733765 (SERCA2), NP_777615 (SERCA3), AAF35375 (SPCA1), NP_000693 (Na^+^/K^+^ ATPase), and AAH31609 (Na^+^/K^+^ ATPase).
(1.73 MB TIF)

Table S1 Progressive hearing loss in litters from *Obl*/+×+/+ matings. Progressive hearing loss detected in *Obl*/+ mutants starts at 1 month of age (6%), with most being deaf by 2 months of age (42%). 50% offspring from *Obl*/+×+/+ mating are expected to show phenotype.
(0.04 MB DOC)

Table S2 Microsatellite markers polymorphic between C3HeB/FeJ and C57BL/6J.
(0.17 MB DOC)

Table S3 Sequencing primers for *Atp2b2*.
(0.07 MB DOC)

Text S1 Supplementary materials.
(0.05 MB DOC)
