# Supplementary figures and images for: The Novel Mouse Mutation Oblivion Inactivates the PMCA2 Pump and Causes Progressive Hearing Loss
Source: PLoS Genet. 2008 Oct 31;4(10):e1000238. doi: 10.1371/journal.pgen.1000238 (PMC2568954; doi:10.1371/journal.pgen.1000238)

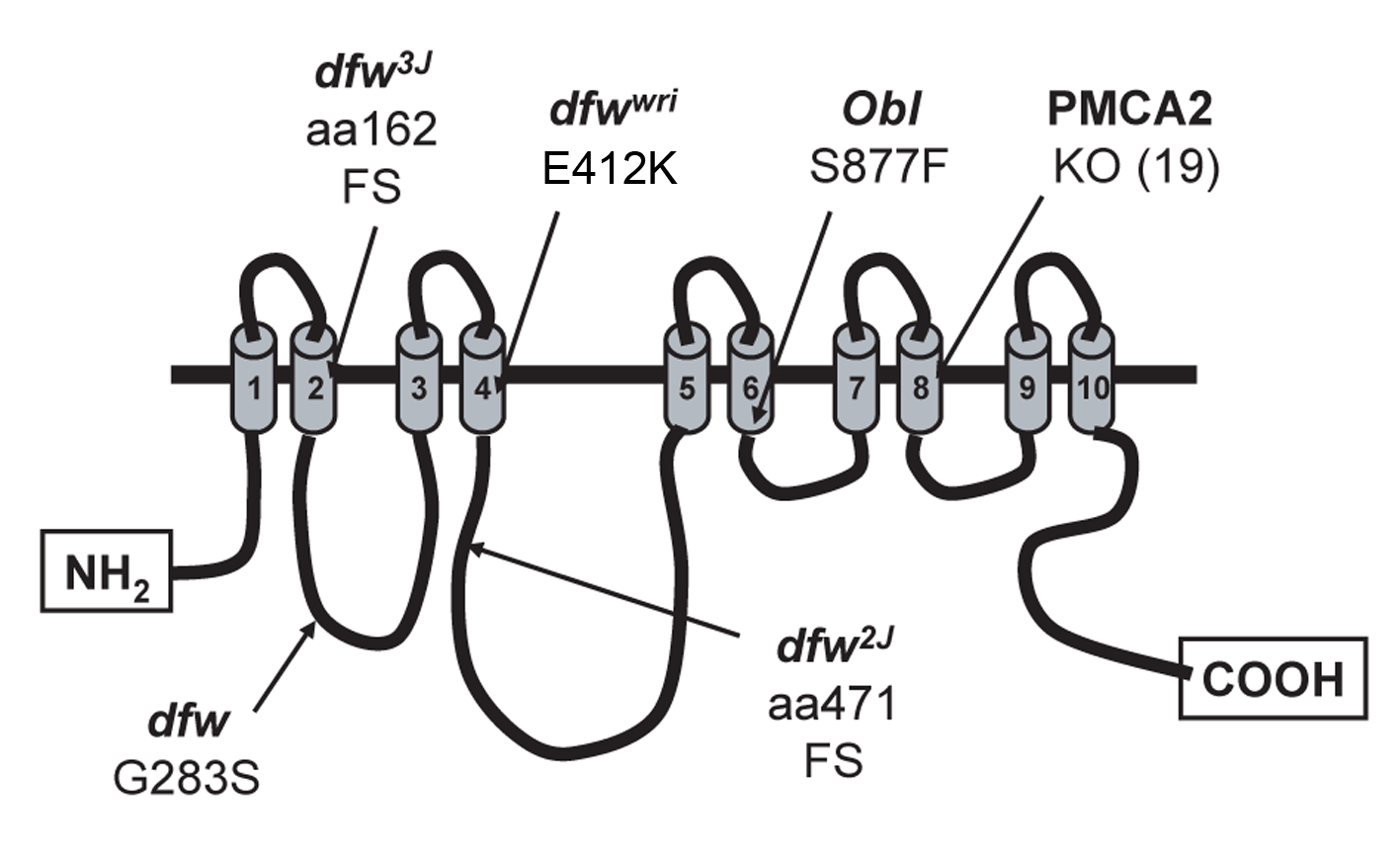

Supplement: Figure S1 — Mutations identified to date affecting PMCA2 protein in mice. Three mutations are missense mutations leading to amino acid substitutions (dfw, wri, Obl), two are small deletions that lead to frame shift mutations and premature truncation of the PMCA2 protein (dfw2J, dfw3J), and one is a targeted null allele (Atp2b2tm1Ges) [8],[13],[21],[26]. Adapted from [21]. (0.29 MB TIF) [file pgen.1000238.s001.tif]

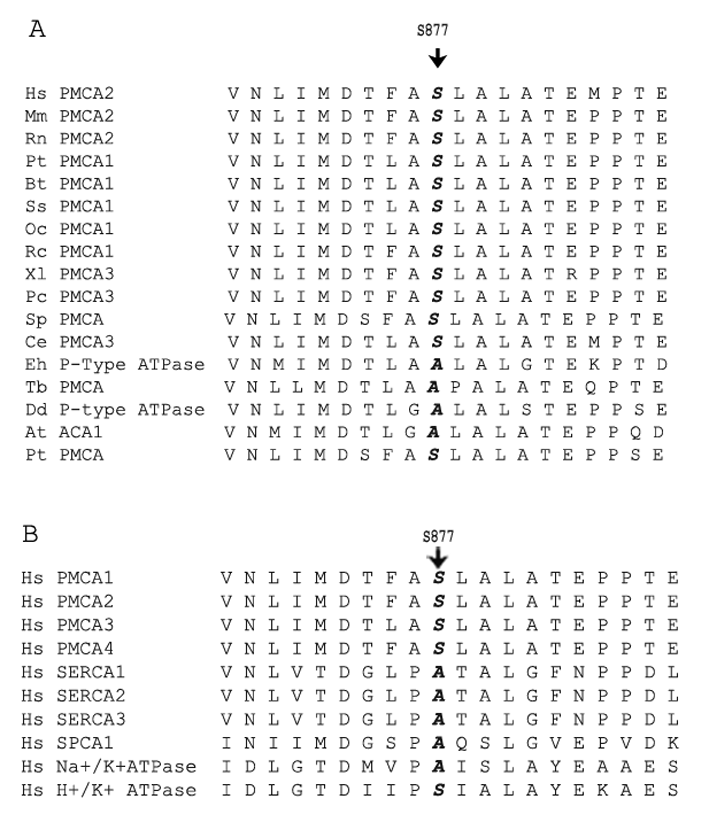

Supplement: Figure S2 — Amino acid alignments of PMCA isoforms. Alignment of the amino acids residues in transmembrane domain 6 of PMCA isoforms in different species (A) and of PMCAs and other P-type ATPases (B). The similarity analysis was performed using the ClustalW program. GenBank accession numbers are listed: NP_001674 (Homo sapiens), Q9ROK7 (Mus musculus), NP_036640 (Rattus norvegicus), XP_509257 (Pan troglodytes), NP_777121 (Bos taurus), NP_999517 (Sus scrofa), Q00804 (Oryctolagus cuniculus), AAK11272 (Rana catesbeiana), AAH77905 (Xenopus laevis), AAR28532 (Procambarus clarkii), AAR13013 (Stylophora pistillata), AAK68551 (Caenorhabditis elegans), XP_653525 (Entamoeba histolytica), AAP46286 (Trypanosoma brucei), EAL62716 (Dictyostelium discoideum), NP_849716 (Arabidopsis thaliana), AAB81284 (Paramecium tetraurelia), NP_001001323 (PMCA1), NP_068768 (PMCA3), NP_001675 (PMCA4), NP_004311 (SERCA1), NP_733765 (SERCA2), NP_777615 (SERCA3), AAF35375 (SPCA1), NP_000693 (Na+/K+ ATPase), and AAH31609 (Na+/K+ ATPase). (1.73 MB TIF) [file pgen.1000238.s002.tif]
