# Supplementary material for: The Novel Mouse Mutation Oblivion Inactivates the PMCA2 Pump and Causes Progressive Hearing Loss
Source: PLoS Genet. 2008 Oct 31;4(10):e1000238. doi: 10.1371/journal.pgen.1000238 (PMC2568954; doi:10.1371/journal.pgen.1000238)
Supplement: Table S1 — Progressive hearing loss in litters from Obl/+×+/+ matings. Progressive hearing loss detected in Obl/+ mutants starts at 1 month of age (6%), with most being deaf by 2 months of age (42%). 50% offspring from Obl/+×+/+ mating are expected to show phenotype. (0.04 MB DOC) [file pgen.1000238.s003.doc]

**Supplementary Table I:**

**Progressive hearing loss in litters from *Obl/+* x +/+ matings**

| **Age (days)** | **Number mice with Preyer reflex** | **Number mice without Preyer reflex** |
| --- | --- | --- |
| 23-25 | 82/82 (100%) | 0/82 (0%) |
| 30-31 | 77/82 (94%) | 5/82 (6%) |
| 37-39 | 58/82 (71%) | 24/82 (29%) |
| 45-46 | 41/66 (62%) | 25/66 (38%) |
| 51-52 | 47/75 (63%) | 28/75 (37%) |
| 58 | 19/33 (58%) | 14/33 (42%) |

Progressive hearing loss detected in *Obl/+* mutants starts at 1 month of age (6%), with most being deaf by 2 months of age (42%). 50% offspring from *Obl/+* X +/+ mating are expected to show phenotype.
