# Supplementary material for: The Novel Mouse Mutation Oblivion Inactivates the PMCA2 Pump and Causes Progressive Hearing Loss
Source: PLoS Genet. 2008 Oct 31;4(10):e1000238. doi: 10.1371/journal.pgen.1000238 (PMC2568954; doi:10.1371/journal.pgen.1000238)
Supplement: Table S2 — Microsatellite markers polymorphic between C3HeB/FeJ and C57BL/6J. (0.17 MB DOC) [file pgen.1000238.s004.doc]

**Supplementary Table II**:

**Microsatellite markers polymorphic between C3HeB/FeJ and C57BL/6J**

| **Microsatellite marker** | **Chromosome** | **Genetic position** |
| --- | --- | --- |
| *D1Mit21* | **1** | 33.89 cM |
| *D1Mit415* | **1** | 53.06 cM |
| *D1Mit445* | **1** | 75.72 cM |
| *D1Mit353* | **1** | 90.34 cM |
| *D2Mit237* | **2** | 28.71 cM |
| *D2Mit128* | **2** | 49.41 cM |
| *D2Mit59* | **2** | 60 cM |
| *D2Mit200* | **2** | 98.36 cM |
| *D3Mit117* | **3** | 2.35 cM |
| *D3Mit339* | **3** | 29.25 cM |
| *D3Mit199* | **3** | 56.1 cM |
| *D4Mit172* | **4** | 11.23 cM |
| *D4Mit58* | **4** | 40.62 cM |
| *D4Mit33* | **4** | 67.6 cM |
| *D4Mit190* | **4** | 75.4cM |
| *D5Mit345* | **5** | 0 cM |
| *D5Mit391* | **5** | 18.3 cM |
| *D5Mit115* | **5** | 40.01 cM |
| *D5Mit168* | **5** | 68.65 cM |
| *D6Mit138* | **6** | 2.35 cM |
| *D6Mit320* | **6** | 22.38 cM |
| *D6Mit366* | **6** | 43.69 cM |
| *D6Mit61* | **6** | 48.16 cM |
| *D6Mit201* | **6** | 62.94 cM |
| *D7Mit178* | **7** | 2.78 cM |
| *D7Mit230* | **7** | 22.45 cM |
| *D7Mit253* | **7** | 42.55 cM |
| *D8Mit190* | **8** | 22.69 cM |
| *D8Mit280* | **8** | 74.11 cM |
| *D9Mit254* | **9** | 20.14 cM |
| *D9Mit214* | **9** | 58.79 cM |
| *D10Mit206* | **10** | 4.47 cM |
| *D10Mit115* | **10** | 32.47 cM |
| *D10Mit12* | **10** | 52.5 cM |
| *D10Mit180* | **10** | 65.22 cM |
| *D11Mit140* | **11** | 25.73 cM |
| *D11Mit35* | **11** | 44.74 cM |
| *D11Mit99* | **11** | 63.21 cM |
| *D12Mit240* | **12** | 3.3 cM |
| *D12Mit69* | **12** | 23.88 cM |
| *D12Mit259* | **12** | 42.92 cM |
| *D12Nds2* | **12** | 62.46 cM |
| *D13Mit3* | **13** | 8.96 cM |
| *D13Mit9* | **13** | 32.44 cM |
| *D13Mit77* | **13** | 50 cM |
| *D14Mit99* | **14** | 1.51 cM |
| *D14Mit260* | **14** | 21.21 cM |
| *D14Mit225* | **14** | 44.1 cM |
| *D15Mit175* | **15** | 5.72 cM |
| *D15Mit43* | **15** | 58.01 cM |
| *D16Mit165* | **16** | 11.17 cM |
| *D16Mit63* | **16** | 30.76 cM |
| *D16Mit152* | **16** | 48 cM |
| *D17Mit113* | **17** | 2.22 cM |
| *D17Mit238* | **17** | 30.64 cM |
| *D18Mit22* | **18** | 8.97 cM |
| *D18Mit187* | **18** | 32.54 cM |
| *D19Mit59* | **19** | 0 cM |
| *D19Mit16* | **19** | 17.16 cM |
| *D19Mit67* | **19** | 35.25 cM |
