# Supplementary material for: The Novel Mouse Mutation Oblivion Inactivates the PMCA2 Pump and Causes Progressive Hearing Loss
Source: PLoS Genet. 2008 Oct 31;4(10):e1000238. doi: 10.1371/journal.pgen.1000238 (PMC2568954; doi:10.1371/journal.pgen.1000238)
Supplement: Table S3 — Sequencing primers for Atp2b2. (0.07 MB DOC) [file pgen.1000238.s005.doc]

**Supplementary Table III.**

**Sequencing primers for *Atp2b2***

| **Primer name** | **Primer sequence (5’-3’)** |
| --- | --- |
| AtpNE1F | TGCCAAGCTCCCTGATGTGC |
| AtpNE1R2 | AGGCTGCCATCAGAACTGGC |
| AtpNE2F | TCCTCACCCAGCAGTTCTAAC |
| AtpNE2R | CTCTACCCATGCAGGTGCAC |
| Atp3F | TGTGGCAGCTCTTGTCTGCTG |
| Atp3R | CCAGATCTAGAAGCATGGAGC |
| Atp4F | CTGTATGCTGTATGCTAGGTAC |
| Atp4R | GCCAGGGACTACACCAGCGTC |
| Atp5F | AGACACCTGTTCCTTCTGTTC |
| Atp5R | GTGGAGTGAACAGATGAATGG |
| Atp6F | CGTTCTGGCCTCTAGGCTAAC |
| Atp6R | GGACCATTGGAATCAAGGTAG |
| Atp7F | CAGAAGCAGTTTAGTGAACATC |
| Atp7R | CTCAGCTGTGGCTGCTTCAG |
| Atp8F | CCAGGCAGAAGGTTAAGAGG |
| Atp8R | CGAGCCAGGTGCTCTGTTATG |
| Atp9F | CAGTGGCTCCTCTGTGGCGAG |
| Atp9R | TGCTTAGATCCAGCCTGCATG |
| Atp10F | TTTGATGACCAAGGTAGGTGG |
| Atp10R | GAAGGATCCTGATACTGTTAC |
| Atp11F | GAAGACCTACCTAAGTGATGC |
| Atp11R | GGCTTCTGTTCAAGCATGCAG |
| Atp12F | TGCTTGAACAGAAGCCAGCTG |
| Atp12R | GAGGAAGCTGCGATGCCATG |
| Atp13F | AGGAAGCCATGCTGAGGCTC |
| Atp13R | GTGTAGTCCAAGCTGACTATC |
| Atp14F | GCCATTCACCTCTCCAGCTGC |
| Atp14R | CAGCCTTTGGTGTGATTCTCC |
| Atp15F2 | TTCTCCCTGCCACTGTCGTAG |
| Atp15R | CGTTGTTGGCCATGGTATGTC |
| Atp16F | CGTTCCACACCTGAGCCGGC |
| Atp16R | GACAGACACGGAGATGGCAC |
| Atp17F | GTGCCATCTCCGTGTCTGTC |
| Atp17R | TCACTTCTAGGACCAATCCTG |
| Atp18F | ATCTTAGCCCTGACCGTTGC |
| Atp18R | GAGGGTGCATTGGAACTACTG |
| Atp19F | GGATCCCAGCCGCCTGCGTGC |
| Atp19R | ATGAGCGTGGCCAGGCGACA |
