# Supplementary material for: The Novel Mouse Mutation Oblivion Inactivates the PMCA2 Pump and Causes Progressive Hearing Loss
Source: PLoS Genet. 2008 Oct 31;4(10):e1000238. doi: 10.1371/journal.pgen.1000238 (PMC2568954; doi:10.1371/journal.pgen.1000238)
Supplement: Text S1 — Supplementary materials. (0.05 MB DOC) [file pgen.1000238.s006.doc]

**Supplementary Materials**

*Complementation test between Obl/+ and dfw/dfw*

To confirm that the missense mutation identified was causative, a complementation test was carried out between *Obl/+* and *dfw/dfw* mice maintained on a C3H/HeJ background [1]. If the Oblivion phenotype is due to mutation of the *Atp2b2* gene, then it would be expected that the two mutations will not complement and the resulting compound heterozygotes from this cross (*Obl/dfw*) would show a phenotype similar to that seen in *Obl* and *dfw* homozygotes. The *dfw* mice used were the original *dfw* allele and homozygotes have a subtle phenotype being deaf and having a slight waddle and head bob [1,2]. The Oblivion homozygote phenotype was much more obvious, with mutants showing severe vestibular dysfunction, falling from side to side, head-tossing, some circling, poor righting reflex and splaying of the hind limbs. This is similar to the phenotype of null alleles of *Atp2b2* in [3,4]. Of the 33 mice that were analysed from this complementation test, 1 (3%) showed mild stargazing; 2 (6%) fell to the side when rearing, one of which also curled in a ball when lifted up and 5 (15%) seemed tottery and fell from side to side when walking and curled up in a ball when lifted up. Since *+/dfw* lose their Preyer reflex by about 1-2 months of age, this test was not a useful discriminator for these mice. Compound heterozygote offspring (*dfw*/*Obl*) showed vestibular phenotypes, intermediate between those seen in the two parental (homozygous) mutants, suggesting that they did not complement and therefore the two mutants are allelic. Only 8/33 (24%) offspring showed non-complementation, which is significantly lower than the 50% that was expected. Since *dfw* and *Obl* both arose on the closely-related genetic backgrounds (C3H/FeJ), it is unlikely that genetic background effects would contribute to this. Another explanation could be that due to the variation in severity of the phenotype, some mutants displaying mild behaviour such as star-gazing were missed. However the presence of a phenotype in *dfw/Obl* compound heterozygotes does support the suggestion that the Oblivion mutant is another allele of the *deafwaddler* locus.

**References**

1. Street VA, McKee-Johnson JW, Fonseca RC, Tempel BL, Noben-Trauth K (1998) Mutations in a plasma membrane Ca^2+^-ATPase gene cause deafness in deafwaddler mice. Nat Genet 19: 390-394.

2. Lane P (1987) New mutants and linkages: deafwaddler (*dfw*). Mouse newsletters 77: 129.

3. Kozel PJ, Friedman RA, Erway LC, Yamoah EN, Liu LH, et al. (1998) Balance and hearing deficits in mice with a null mutation in the gene encoding plasma membrane Ca^2+^-ATPase isoform 2. J Biol Chem 273: 18693-18696.

4. Takahashi K, Kitamura K (1999) A point mutation in a plasma membrane Ca^2+^-ATPase gene causes deafness in Wriggle Mouse Sagami. Biochem Biophys Res Commun 261: 773-778.
